# Distinct microglial response against Alzheimer's amyloid and tau pathologies characterized by P2Y12 receptor

**DOI:** 10.1093/braincomms/fcab011

**Published:** 2021-01-29

**Authors:** Jun Maeda, Takeharu Minamihisamatsu, Masafumi Shimojo, Xiaoyun Zhou, Maiko Ono, Yukio Matsuba, Bin Ji, Hideki Ishii, Masanao Ogawa, Hiroyasu Akatsu, Daita Kaneda, Yoshio Hashizume, John L Robinson, Virginia M -Y Lee, Takashi Saito, Takaomi C Saido, John Q Trojanowski, Ming-Rong Zhang, Tetsuya Suhara, Makoto Higuchi, Naruhiko Sahara

**Affiliations:** 1 Department of Functional Brain Imaging, National Institute of Radiological Sciences, National Institutes for Quantum and Radiological Science and Technology, Chiba, Japan; 2 Laboratory for Proteolytic Neuroscience, RIKEN Center for Brain Science, Saitama, Japan; 3 Department of Advanced Nuclear Medicine Science, National Institute of Radiological Sciences, National Institutes for Quantum and Radiological Science and Technology, Chiba, Japan; 4 Department of Neuropathology, Choju Medical Institute, Fukushimura Hospital, Aichi, Japan; 5 Department of Community-based Medical Education, Nagoya City University Graduate School of Medical Sciences, Aichi, Japan; 6 Center for Neurodegenerative Disease Research, University of Pennsylvania Perelman School of Medicine, Philadelphia, PA 19104-2674, USA; 7 Department of Neurocognitive Science, Institute of Brain Science, Nagoya City University Graduate School of Medical Sciences, Aichi, Japan

**Keywords:** Alzheimer’s disease, microglia, P2Y12 receptor, tauopathy, amyloid pathology

## Abstract

Microglia are the resident phagocytes of the central nervous system, and microglial activation is considered to play an important role in the pathogenesis of neurodegenerative diseases. Recent studies with single-cell RNA analysis of CNS cells in Alzheimer’s disease and diverse other neurodegenerative conditions revealed that the transition from homeostatic microglia to disease-associated microglia was defined by changes of gene expression levels, including down-regulation of the P2Y12 receptor gene (*P2Y12R*). However, it is yet to be clarified in Alzheimer’s disease brains whether and when this down-regulation occurs in response to amyloid-β and tau depositions, which are core pathological processes in the disease etiology. To further evaluate the significance of P2Y12 receptor alterations in the neurodegenerative pathway of Alzheimer’s disease and allied disorders, we generated an anti-P2Y12 receptor antibody and examined P2Y12 receptor expressions in the brains of humans and model mice bearing amyloid-β and tau pathologies. We observed that the brains of both Alzheimer’s disease and non-Alzheimer’s disease tauopathy patients and tauopathy model mice (rTg4510 and PS19 mouse lines) displayed declined microglial P2Y12 receptor levels in regions enriched with tau inclusions, despite an increase in the total microglial population. Notably, diminution of microglial immunoreactivity with P2Y12 receptor was noticeable prior to massive accumulations of phosphorylated tau aggregates and neurodegeneration in rTg4510 mouse brains, despite a progressive increase of total microglial population. On the other hand, Iba1-positive microglia encompassing compact and dense-cored amyloid-β plaques expressed P2Y12 receptor at varying levels in amyloid precursor protein (APP) mouse models (APP23 and *App^NL-F/NL-F^* mice). By contrast, neuritic plaques in Alzheimer’s disease brains were associated with P2Y12 receptor-negative microglia. These data suggest that the down-regulation of microglia P2Y12 receptor, which is characteristic of disease-associated microglia, is intimately associated with tau rather than amyloid-β pathologies from an early stage and could be a sensitive index for neuroinflammatory responses to Alzheimer’s disease-related neurodegenerative processes.

## Teaser

Homeostatic microglial marker P2Y12 receptor was accumulated around amyloid plaques in mouse models; this receptor was reduced in tauopathy mouse models prior to massive tangle formation. The down-regulation of P2Y12 receptor could be a sensitive index for neuroinflammatory responses to tau-induced neurodegeneration.

## Graphical Abstract

**Graphical Abstract:**
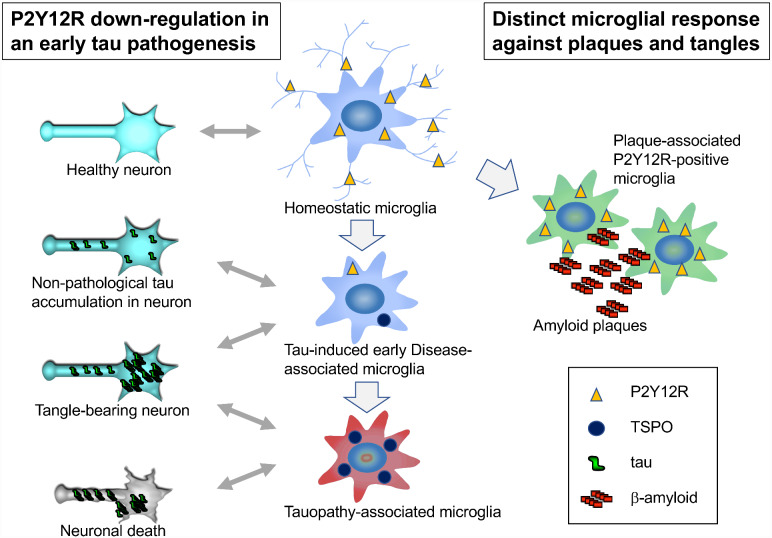

## Introduction

Neurodegenerative diseases (e.g. Alzheimer’s disease (AD), Parkinson’s disease, frontotemporal lobar degeneration, Huntington’s disease, amyotrophic lateral sclerosis) are clinically defined by a substantial decline of cognitive and/or sensorimotor functions and comorbid psychiatric conditions, along with the disease-associated neuropathological processes. These processes are commonly linked to age-related protein aggregation termed proteinopathy. In particular, AD is neuropathologically diagnosed by the presence of extracellular senile plaques composed of amyloid-β (Aβ) peptides and intracellular neurofibrillary tangles (NFTs) consisting of bundles of paired helical filaments of the microtubule-associated protein tau. The molecular mechanisms that link these protein aggregations with neurodegeneration remain elusive. On the other hand, neuroinflammation has been suggested to play important roles in neurodegenerative disease progression. In fact, it is reported that activated astrocytes and microglia are closely associated with Aβ and tau pathologies (Leyns and Holtzman, 2017). In a normal condition, microglia are implicated in the phagocytosis and degradation of misfolded protein species. Chronic neuroinflammation could be induced by activated microglia through the release of multiple neurotoxic factors (e.g. tumor necrosis factor-α, nitric oxide, interleukin-1β (IL-1β), and reactive oxygen species) in response to protein aggregates, ultimately leading to neurodegeneration (Lull and Block, 2010).

Microglial activation is classified into two categories, pro-inflammatory classical activation and anti-inflammatory alternative activation (Colton, 2009). Although the pro/anti-inflammatory classification is well defined by the different functional states of microglia, transcriptome analysis of microglia derived from models of neurodegenerative diseases failed to show a distinguishable pro-inflammatory or anti-inflammatory signature (Chiu *et al.*, 2013; Holtman *et al.*, 2015; Keren-Shaul *et al.*, 2017; Friedman *et al.*, 2018). Meanwhile, disease-associated microglia (DAM) represent a new category of microglia (Keren-Shaul *et al.*, 2017), which revealed a reduction in the expressions of 68 homeostatic microglial genes as well as up-regulation of 28 inflammatory molecules (Krasemann *et al.*, 2017). Single-cell RNA-seq analysis revealed that DAM exhibited increased expressions of *APOE*, *TREM2*, and *Cst7*, but decreased expressions of homeostatic genes P2Y12 receptor *(P2Y12R)* and *CX3CR1* in a mouse model of Aβ pathologies dubbed 5XFAD (Keren-Shaul *et al.*, 2017). These DAM phenotypes are mostly derived from AD models with engineered amyloid precursor protein (*APP*) and related genes leading to Aβ plaque formation. In AD patients, however, a recent study pointed out differences in the expression of DAM phenotypes in comparison with APP-based mouse models (Navarro *et al.*, 2018). Phenotypic changes of microglia accompanied by a loss of anti-inflammatory activities may exist along with the advancement of Aβ accumulations in human brains.

To investigate the link between microglial activation and tau pathology, the tauopathy mouse model PS19 with TREM2 KO was examined (Leyns *et al.*, 2017), demonstrating a reduction of brain atrophy and a decrease in DAM-associated markers, *APOE* and *Cst7*, and tau-induced neurodegeneration by the lack of TREM2. This indicates that DAM phenotypes were actively engaged in the cause of tauopathy. Recent studies used in vivo imaging to examine microglial activation during the neurodegenerative process. The mitochondrial 18-kDa translocator protein (TSPO), which is expressed on activated microglia, astrocytes and infiltrating immune cells in the central nervous system (CNS), is one of the inflammatory markers. Previously, we established mitochondrial TSPO imaging in living AD mouse models to assess microglial activation (Ji *et al.*, 2008; Maeda *et al.*, 2011; Ishikawa *et al.*, 2018). We demonstrated longitudinal in vivo monitoring of tau pathology and TSPO accumulation in tauopathy models, PS19 and rTg4510 mice, using small-animal PET imaging (Maeda *et al.*, 2011; Ishikawa *et al.*, 2018). Our findings of the age-dependent TSPO accumulation along with pathological tau accumulation and brain atrophy suggested that DAM phenotype might be involved in tau-induced neurodegeneration.

As a counterpart of the DAM status, homeostatic microglia exist in the adult brain under non-disease condition. Homeostatic microglial markers can be identified using gene and microRNA array analysis and quantitative proteomic analysis (Butovsky *et al.*, 2014). Several studies confirmed the decrease of homeostatic genes (e.g. *Olfml3, Fcrls, Tmem119, Siglech, Gpr34, P2Y12R*) under inflammatory conditions. Among them, the metabotropic purinergic receptor P2Y12R is one of the specific markers for detecting homeostatic microglia and is highly sensitive to neuroinflammatory changes (Amadio *et al.*, 2014; Mildner *et al.*, 2017; Zrzavy *et al.*, 2017). However, it is yet to be determined how the emergence of DAM in response to Aβ and tau pathologies is associated with the downregulation of P2Y12R. It is also necessary to examine whether P2Y12R declines are implicated in neurodegenerative etiology from an early stage. Moreover, the significance of P2Y12R as a target molecule for in vivo imaging supplementary to TSPO also remains elusive. To address these issues, we generated an anti-P2Y12R antibody to detect either human or murine P2Y12R and examined P2Y12R expressions in the brains of AD and non-AD tauopathy patients and tau and APP mouse models. Furthermore, we radiosynthesized a P2Y12R ligand, [^11^C]AZD1283, and attempted to apply it to in vitro autoradiography for the purpose of exploring the possibility of capturing disease-related changes of P2Y12R by a PET probe.

## Materials and methods

### Antibodies

Rabbit polyclonal antibodies for human or mouse P2Y12 receptors were raised against c-terminal polypeptides corresponding to amino acid residues 324–342 (QDNRKKEQDGGDPNEETPM) and 329–347 (GTNKKKGQEGGEPSEETPM), respectively. These c-terminal sequences are less homologous between human and mouse, and antigenicity of these epitopes are predicted by accessibility, surface probability and hydrophilicity. Obtained antisera were purified by immune-affinity columns. Monoclonal antibodies AT8 (MN1020, mouse monoclonal, Thermo Fisher Scientific, Waltham, MA), TSPO (EPR5384, rabbit monoclonal, Abcam, Cambridge, UK), GFAP (2.2B10, rat monoclonal, Zymed, San Francisco, CA), Iba1 (MABN92, mouse monoclonal, Merck Millipore, Burlington, MA), rabbit polyclonal antibody Iba1 (019-19741, Wako, Japan) and β-actin (A1987, mouse monoclonal, Merck) were used for immunohistochemistry.

### Human tissue

Postmortem human brains were obtained from the University of Pennsylvania Center for Neurodegenerative Disease Research and Fukushimura Hospital. Frontal and temporal cortices were examined from each brain specimen. Amyloid histopathology was assessed according to CERAD criteria (Mirra *et al.*, 1991). NFT pathology was staged according to Braak and Braak (Braak and Braak, 1991). Cases of senile dementia of the NFT type (SD-NFT), a subset of dementia characterized by numerous NFTs in the hippocampal region and the absence of amyloid plaques throughout the brain, were diagnosed by these criteria (Bancher *et al.*, 1997; Yamada, 2003). All procedures involving the use of human materials were performed in accordance with the ethical guidelines of the Institutional Review Boards (IRBs) of the University of Pennsylvania, Fukushimura Hospital and the National Institutes for Quantum and Radiological Science and Technology. The Institutional Animal Care Committee of the National Institutes for Quantum and Radiological Science and Technology and the RIKEN institute approved all animal study protocols.

### Mice

Two lines of tauopathy mouse models (rTg4510 and PS19) and two lines of APP mouse models (APP23 and *App^NL-F/NL-F^*) were examined in this study. To generate rTg4510 mice, the parental P301L mutated human tau responder line (tetO-MAPT*P301L, FVB/N background) and the parental tTA activator line (Camk2a-tTA, 129/SV background) were maintained as previously described (Santacruz *et al.*, 2005; Ishikawa *et al.*, 2018). The rTg4510 mice were maintained on a standard diet lacking doxycycline to present a lifetime expression of transgenic human tau. PS19 mice expressing the P301S mutant human tau under the control of mouse prion promoter (Yoshiyama *et al.*, 2007), APP23 mice expressing human APP with the Swedish mutation (Sturchler-Pierrat *et al.*, 1997), and *App^NL-F/NL-F^* mice that produce a humanized Aβ peptide by changing three amino acids (G676R, F681Y and H684R) (Saito *et al.*, 2014) were maintained on a C57BL/6 background. These mice were housed with *ad libitum* food and water in their cages at 25 °C in a 12-hr light/dark cycle. All experiments were performed in accordance with the institutional guidelines on use of laboratory animals and were approved by the National Institutes for Quantum and Radiological Science and Technology and RIKEN Institutional Animal Care and Use Committees.

### Tissue extraction and western blot

Mouse brain homogenates were obtained from rTg4510 mice at 2–6 months of age (male, *n* = 5) and from non-transgenic mice at 2–6 months of age (male, *n* = 5) according to the previous report (Shimojo *et al.*, 2020). Briefly, forebrains including cerebral cortices and hippocampi were homogenized in 20 mM HEPES-NaOH, pH7.4, 150 mM NaCl, 2 mM EDTA supplemented with protease inhibitor cocktail (Roche) and phosphatase inhibitors (Sigma) by tissue grinder, and lysed by the addition of SDS-PAGE sample buffer with further resuspension using a 26 G needle. The extracted proteins were separated by SDS-PAGE with pre-cast 10% Tris-glycine sodium dodecyl sulfate-polyacrylamide gel electrophoresis (SDS-PAGE) gels (Nakarai, Japan) and transferred onto nitrocellulose membranes (BioRad Laboratories, Hercules, CA). After blocking with 5% Skim Milk (in TBS with 0.05% Tween-20), the membranes were incubated with anti-mouse P2Y12R antibody (1:5000) with or without antigen peptide (final concentration 1 µg/mL). Secondary antibody, peroxidase-conjugated goat anti-rabbit antibody (1:5000; 111-035-144, Jackson ImmunoResearch, West Grove, PA), was reacted with membranes and visualized by Amersham Imager 600 (Cytiva, Marlborough, MA) using the enhanced chemiluminescence system (ECL PLUS kit; PerkinElmer). Quantitative analysis was performed with Image Studio^TM^ Lite software (LI-COR, Lincoln, NE).

### Fluorescence staining

Formalin-fixed brain sections were paraffin-embedded and cut into 4–5-µm sections. In vitro fluorescence staining of human brain sections was performed with 1-fluoro-2,5-bis(3-hydroxycarbonyl-4-hydroxystyryl)benzene (FSB; Dojindo Laboratory, Japan). Deparaffinized sections were incubated with 20 µg/mL FSB in 50% ethanol for 30 min at 25 °C, followed by washing with 50% ethanol for 5 min. The process was continued for immunofluorescence staining. Sections were autoclaved for antigen retrieval followed by blocking with TNB blocking solution (PerkinElmer, Waltham, MA). hP2Y12R (1:5000), mP2Y12R (1:5000), GFAP (1:500), TSPO (1000), Iba1 (Wako: 1000; Merck Millipore: 1:100) and AT8 (1:250) were used for primary antibodies. For fluorescence images, Alexa Fluor 488 goat anti-mouse IgG (A32727), Alexa Fluor 555 goat anti-mouse IgG (A32727), Alexa Fluor 488 goat anti-rabbit IgG (A32731), Alexa Fluor 555 goat anti-rabbit IgG (A32732) and Alexa Fluor 488 goat anti-rat IgG antibodies 1:500 (A11006, Thermo Fisher Scientific) were used. For signal amplification, biotinylated goat anti-rabbit IgG (1:1000; AP187B, Merck Millipore) was used as a secondary antibody, and immunoreactivity was visualized using fluorescein tyramide or tetramethylrhodamine signal amplification (TSA fluorescein system, PerkinElmer). For co-labeling with mP2Y12R and Iba1 (Wako; rabbit polyclonal) antibodies, sections were first incubated with Iba1 antibody (1:2500), followed by incubation with biotinylated goat anti-rabbit IgG (1:1000). After labeling with TSA fluorescein, sections were incubated with mP2Y12R antibody (1:5000) and labeled with Alexa Fluor 555 goat anti-rabbit IgG antibody (1:500). To avoid bleed-through of FSB fluorescence into GFP filter, some of the sections were stained and immune-labeling images were captured, followed by incubation with FSB and the identical fields were re-captured. Images were captured by fluorescence microscope (BZ-X700, Keyence, Japan). For image quantification of P2Y12R-positive signals, the area of the P2Y12R-positive signal was extracted and quantified by BZ-X Analyzer (Keyence). The P2Y12R-positive level was displayed as the signal ratio (%) to the total area (0.62 ± 0.044 or 1.2 ± 0.052 mm^2^ field of CA1 or motor cortex from each rTg4510 brain section, respectively).

### Radiochemical synthesis

[^11^C]CO_2_ is produced by ^14^N(p, α)^11^C nuclear reaction using a CYPRIS HM-18 cyclotron (Sumitomo Heavy Industry, Japan). [^11^C]CO_2_ (average 614 GBq, *n* = 3) was concentrated in a stainless tube at −100°C. Then, after heating the stainless tube, concentrated [^11^C]CO_2_ was reduced to [^11^C]CH_4_ by passing through a Ni column at 400 °C under H_2_ gas flow (50 mL/min). The generated [^11^C]CH_4_ was collected in a Porapak Q trap at −196°C. Then the [^11^C]CH_4_ was released by a stream of nitrogen gas containing 5% NH_3_ (500 mL/min), and the mixed gas was passed over a heated quartz column, which was staffed with Pt wire (1.3 g), at 1000 °C. The generated [^11^C]NH_4_CN was trapped by bubbling through a solution (DMF) of reaction mixture containing copper iodide (1.4 mg, 7.4 µmol) and ethyl 6-(4-((benzylsulfonyl)carbamoyl)piperidin-1-yl)-5-bromo-2-methylnicotinate (1.4 mg, 2.7 µmol) at room temperature. Then, the reaction mixture was heated at 180 °C. After 5 min, the reaction mixture was transferred to a preparative HPLC system. HPLC separation was performed on a COSMOSIL Cholester (10 mm i.d. × 250 mm) using CH_3_CN/100 mM ammonium acetate (4/6, v/v) at 5 mL/min, and detected at UV 280 nm. The radioactive fraction corresponding to [^11^C]AZD1283 (Rt = 10 min) was collected in a ﬂask, evaporated to dryness under reduced pressure, re-dissolved in 3 mL of sterile saline with 0.1% Tween 80, and passed through a 0.22 μm Millipore ﬁlter to give 2.6 GBq of [^11^C]AZD1283 (average *n* = 3) with a total synthesis time of 32 min (average *n* = 3). The identity of [^11^C]AZD1283 (Rt = 5.8 min) was conﬁrmed by comparison with an authentic sample (COSMOSIL Cholester (4.6 mm i.d. × 250 mm) using MeCN/100 mM ammonium acetate (6/4, v/v) at 1 mL/min, UV 280 nm). Molar activity (Am) was also determined by analytical HPLC in a range of 40 - 105 GBq/µmol.

### In vitro [^11^C]AZD1283 autoradiography

PS19 (*n* = 15) and non-transgenic (non-tg; *n* = 5) mice at 6–12 months of age (*n* = 15) for the tauopathy mouse model, and *App^NL-F/NL-F^* (*n* = 8) and wild-type (*n* = 8) mice at 12–13 months of age for the amyloid model mouse were anesthetized with 1.5% (v/v) isoflurane and trans-cardially perfused with PBS. Brains were removed and quickly frozen. Brain samples were cut into 20-µm thick coronal slices by cryotome (HM560; Carl Zeiss, Oberkochen, Germany), and the slices were mounted on glass slides (Matsunami Glass, Japan) and stored at 4 °C pending assays. These sections were reacted with 1 nM [^11^C]AZD1283 in 50 mM Tris-HCL buffer, pH 7.4, at room temperature for 1 h, washed with ice-cold Tris-HCl buffer for 2 min twice, warmly blow-dried, and contacted to an imaging plate (BAS-MS; Fuji Film, Japan) for 2 h. The imaging plate data were scanned with a BAS5000 system (Fuji Film).

### Statistical analysis

Statistical analysis was conducted using PRISM7 (GraphPad Software Inc., La Jolla, CA). For comparison of two groups, data were analyzed by student *t*-test. For multiple comparisons between control and other groups, one-way ANOVA followed by Dunnett post hoc was performed. For multiple comparisons involving multiple variables (e.g. genotype, region of interest), two-way ANOVA followed by Bonferroni’s comparisons test was performed. Statistical significance was determined by *P*-value < 0.05.

### Data availability

The authors confirmed that the data supporting the findings of this study are available upon request. The antibodies generated in our laboratory are available to collaborators.

## Results

### Reduction of P2Y12R-positive microglia in human AD brains

**Figure 1:**
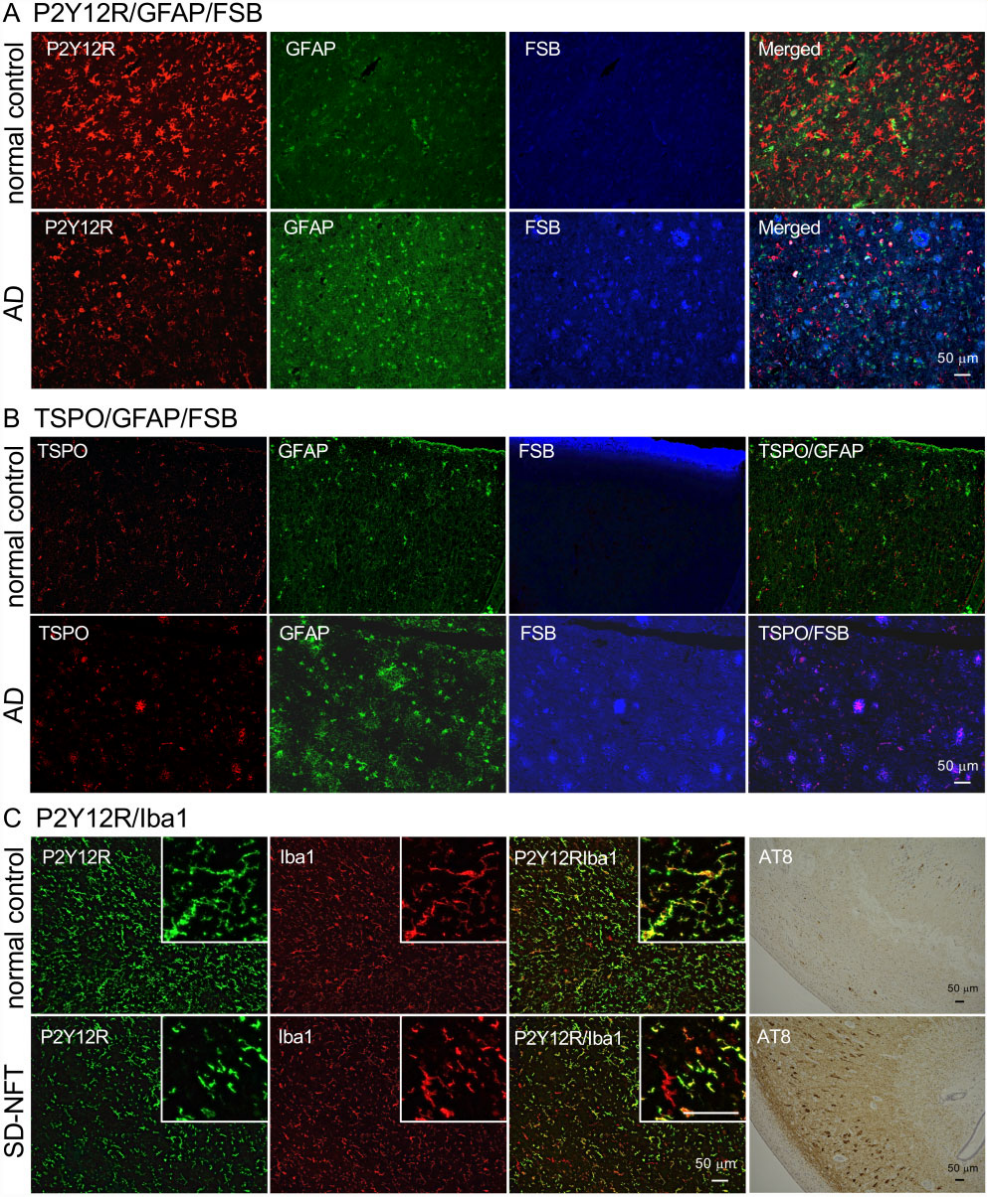
**Fluorescence labeling of anti-glial protein antibodies and β-sheet-binding dye in human brain sections.** **A**. Co-labeling of P2Y12R, GFAP and FSB in normal control (78-year-old, female, non-demented subject, Braak NFT: I, CERAD plaque: 1) and AD (73-year-old, male, Braak NFT: III, CERAD plaque: 3) temporal cortices. **B**. Co-labeling of TSPO, GFAP, and FSB in normal control (96-year-old, female, non-demented subject, Braak NFT: II, CERAD plaque: 0) and AD (47-year-old, female, Braak NFT: III, CERAD plaque: 3) temporal cortices. C. Double-immunofluorescence staining of anti-P2Y12R and anti-Iba1 (mouse monoclonal) antibodies in normal control (normal control subject in B) and SD-NFT (96-year-old, female, Braak NFT: III, CERAD plaque: 1) hippocampal sections. Inboxes show P2Y12R/Iba1 double-positive and P2Y12R-negative/Iba1-positive cells and cell processes. Serial sections of each human brain were DAB stained by AT8 antibody. Scale bars = 50 µm.

P2Y12R is a Gi-coupled receptor for adenosine diphosphate (ADP) initially identified on platelets (Hollopeter *et al.*, 2001). It was reported that an anti-P2Y12R antibody labeled parenchymal microglia in human central nervous system (CNS) tissues, with the staining being decreased in the brains derived from multiple sclerosis and AD cases (Mildner *et al.*, 2017). In this study, an anti-human P2Y12R antibody was generated by immunization of rabbits with the peptide of the c-terminal region. The immunohistochemical examination demonstrated that this antibody labeled microglia that were distinct from GFAP staining (Fig. 1A). In the AD brain, the number of P2Y12R-positive microglia was less than that of the healthy control brain, and some of the P2Y12R-positive microglia were transformed into an amoeboid shape (Fig. 1A, AD P2Y12R image). A β-sheet-binding dye, FSB, labeled NFTs, and amyloid plaques in AD, but the P2Y12R immunofluorescence signal was not clearly associated with these deposits (Fig. 1A, AD merged image). In contrast to P2Y12R reduction, GFAP- and TSPO-positive signals were increased in AD sections without overlapping with each other (Fig. 1A and B). Taken together with previous findings on accumulations of astrocytes and TSPO-positive microglia in the AD brain (Maeda *et al.*, 2011; Ji *et al.*, 2015; Leyns and Holtzman, 2017), the current data provide evidence for the glial activation induced by AD pathologies accompanying notable P2Y12R declines. To confirm that the anti-human P2Y12R antibody recognized microglia, double-immunostaining with anti-human P2Y12R and Iba1 antibodies was performed for the hippocampal sections of a healthy control and a patient with senile dementia of NFT type (SD-NFT), a subset of dementia characterized by numerous NFTs in the hippocampal region and absence of amyloid plaques throughout the brain. Most Iba1-positive microglial cells were co-labeled with anti-human P2Y12R antibody in healthy control subjects, while a significant subset of Iba1-positive microglia was P2Y12R-negative in SD-NFT subjects (Fig. 1C inboxes and Supplemental Fig. 1).

### Reduction of P2Y12R-positive microglia in rTg4510 mouse brains

**Figure 2:**
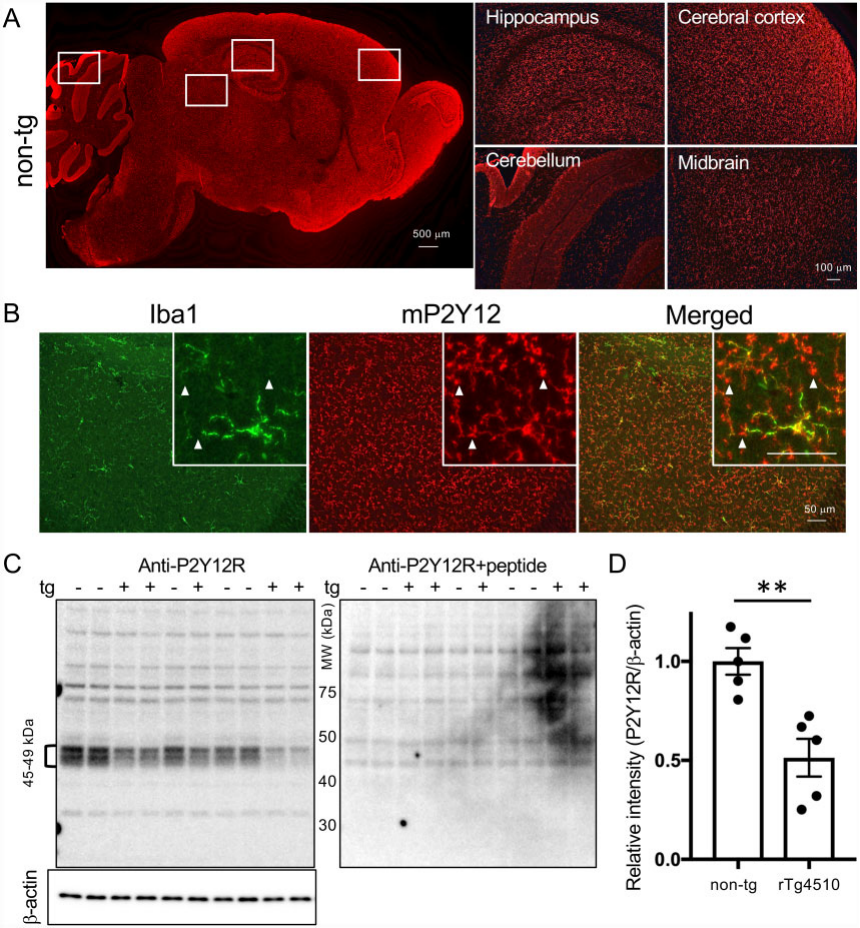
**Detection of mouse P2Y12R and quantification of P2Y12R signals.** **A.** Mouse P2Y12R immunoreactivity in a sagittal brain section from 2-month-old non-tg mouse (left panel). Scale bar = 500 µm. Higher magnifications of P2Y12R-positive signals in hippocampus, cerebral cortex, cerebellum and midbrain (right panels). Scale bars = 100 µm. **B**. Double-immunofluorescence staining of anti-P2Y12R and anti-Iba1 (rabbit polyclonal) antibodies in wild-type mouse cerebral cortex (8-month-old). Arrowheads in inboxes show P2Y12R-positive processes of microglia cells, which are negative for anti-Iba1 antibody. Scale bar = 50 µm. **C**. Western blot analysis of P2Y12R and β-actin proteins. 45-49 kDa bands (49 kDa was major band) were absorbed by the antigen treatment. D. Quantitative data of P2Y12R levels normalized by β-actin levels. rTg4510 mice (2–6-month-old, *n* = 5) have significantly less mouse P2Y12R protein than non-tg littermates (*n* = 5). Values are mean ± SEM. Mean value of non-tg mice was set as 1. **P < 0.01 (student *t*-test).

Antigen peptide of the c-terminal region of mouse P2Y12R, with its amino acid sequence being distinct from that of humans, was synthesized and used for the immunization of rabbits. Immunofluorescence staining of anti-mouse P2Y12R antibody showed intense signals in the cerebral cortex, hippocampus, and olfactory bulb, and weaker signals in the cerebellum and midbrain in 2-month-old non-tg mouse brain (Fig. 2A). Double immunofluorescence staining for P2Y12R and Iba1 demonstrated double-positive microglia in the wild-type mouse brain (Fig. 2B). Numerous foot processes of microglia were immunolabeled with the P2Y12R antibody, but not with the Iba1 antibody (Fig. 2A inboxes). To test this P2Y12R antibody by western blotting, brain extracts containing plasma membranes from 2–6-month-old rTg4510 and non-tg littermates were applied to SDS-PAGE. Among a wide range of bands, there were individual variations in signal intensities of 45-49 kDa bands (Fig. 2C). Since a molecular mass of P2Y12R protein was predicted as around 50 kDa (Amadio *et al.*, 2014), these bands were expected to be P2Y12R proteins. Antibody absorption test by antigen peptide confirmed the elimination of 45–49 kDa bands in both rTg4510 and non-tg brain extracts (Fig. 2C). When 45–49 kDa bands were quantitated and compared between rTg4510 and non-tg mice, the signal intensity in rTg4510 mice was significantly less than that in non-tg mice, suggesting decreased homeostatic microglia in the rTg4510 mice (Fig. 2D).

### Decline of P2Y12R in rTg4510 mouse brains from young ages

**Figure 3:**
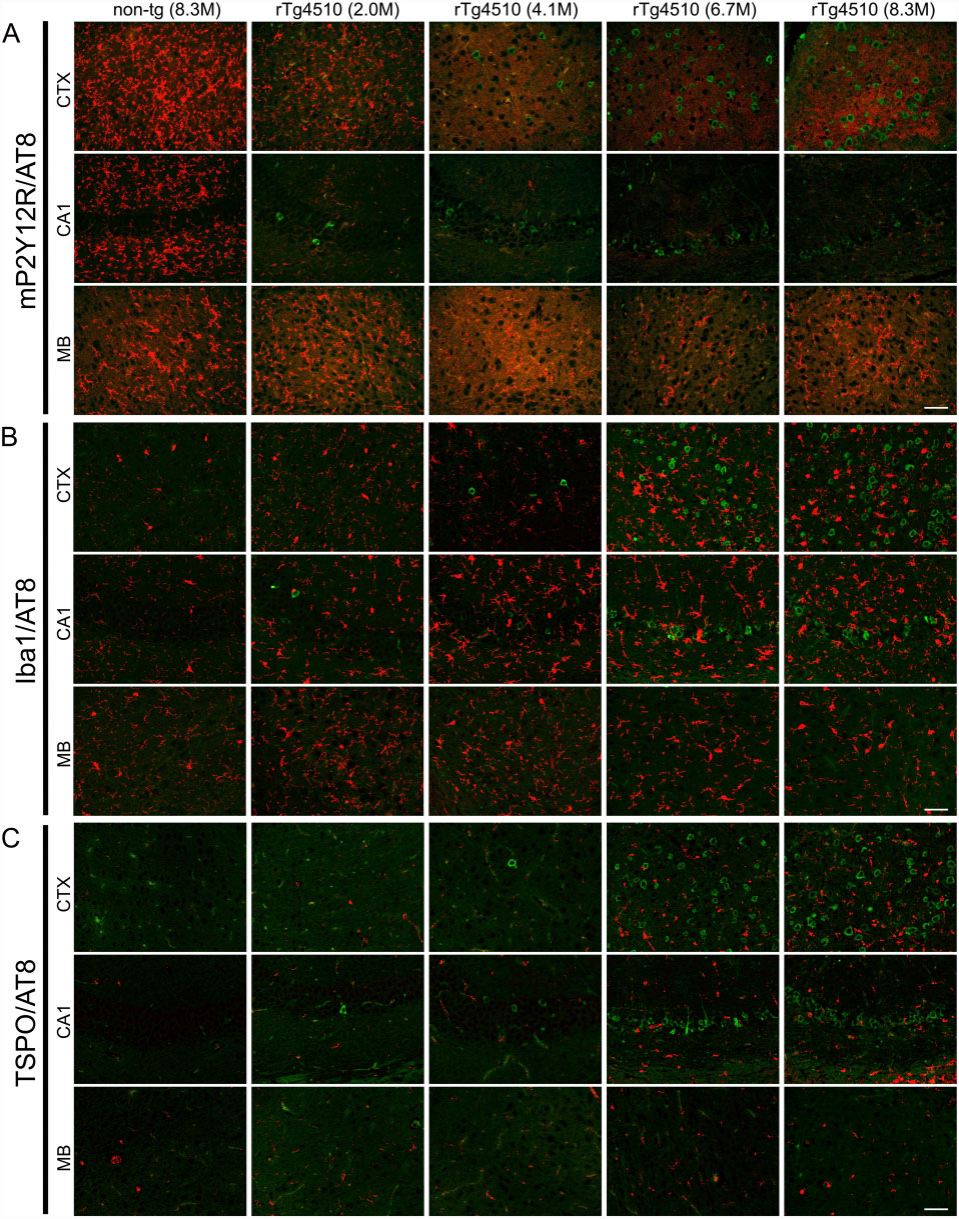
**Temporal changes of microglia markers in rTg4510 brains.** **A**. Double-immunofluorescence staining of anti-P2Y12R (red) and anti-phospho-tau (AT8; green) antibodies in cerebral cortex (CTX), hippocampal CA1 (CA1), and midbrain (MB) of non-tg (male 8.3-month-old) and rTg4510 (female 2-, 4.1-, 6.7-, and male 8.3-month-old) mice. The decrease of P2Y12R immunostaining appeared in CTX and CA1 of rTg4510 mice as early as 2 months of age. **B**. Double-immunofluorescence staining of anti-Iba1 (red) and AT8 (green) antibodies in CTX, CA1, and MB of non-tg (8.3-month-old) and rTg4510 (2-, 4.1-, 6.7-, and 8.3-month-old) mice. Iba1 immunoreactivity was increased with the accumulation of AT8-positive intracellular tau inclusions. C. Double-immunofluorescence staining of anti-TSPO (red) and AT8 (green) antibodies in CTX, CA1, and MB of non-tg (8.3-month-old) and rTg4510 (2-, 4.1-, 6.7-, and 8.3-month-old) mice. TSPO immunoreactivity was increased with the accumulation of AT8-positive intracellular tau inclusions. Scale bars = 50 µm.

**Figure 4:**
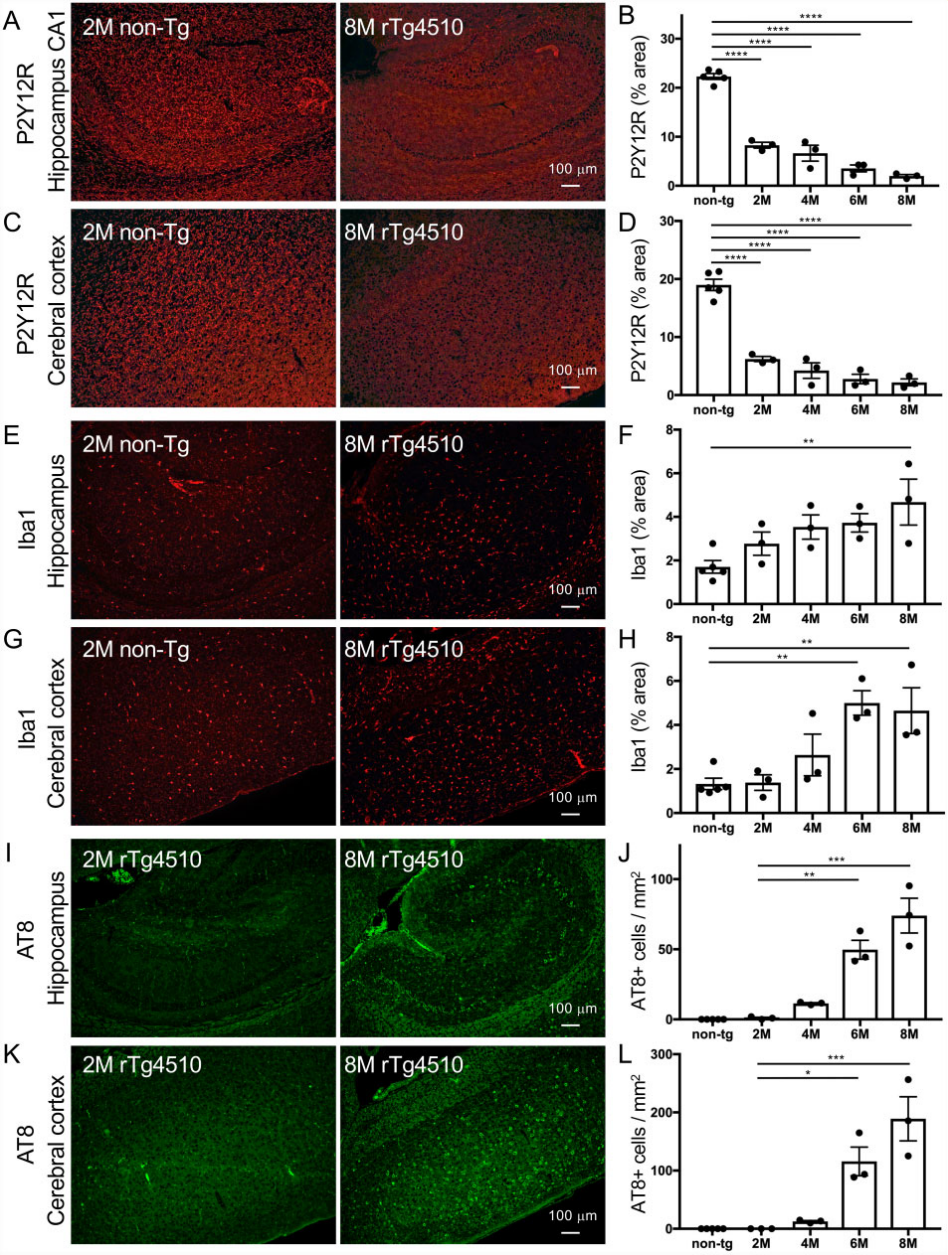
**Quantitative analyses for P2Y12R, Iba1 and AT8 immunofluorescence staining in non-tg and rTg4510 mouse brains.** Total 17 mice (non-tg mice: 2-, 4.1-, 4.9-, 6.7-, and 8.3-month-old, 3 males and 2 females; rTg4510 mice: 1.5 to 2- (2 males and 1 female), 4.1 to 4.5- (1 male and 2 females), 5.9 to 6.7- (1 male and 2 females), 7.7 to 8.3-month-old (3 males), *n* = 3 each) were examined. **A**. Representative P2Y12R immunofluorescence labeling images in hippocampal areas of non-tg (2-month-old) and rTg4510 (8-month-old) mice. Scale bar = 100 µm. **B**. Semi-quantification of P2Y12R signals in hippocampal CA1 from non-tg and rTg4510 mice. Positive signals of cells and processes are presented as % areas. Values are mean ± SEM. The difference between control and other groups is significant by one-way ANOVA (*P* < 0.0001). **C**. Representative P2Y12R immunofluorescence labeling images in motor cortices of non-tg (2-month-old) and rTg4510 (8-month-old) mice. Scale bar = 100 µm. **D**. Semi-quantification of P2Y12R signals in motor cortices from non-tg and rTg4510 mice. Positive signals of cells and processes are presented as % areas. Values are mean ± SEM. ****P < 0.0001, versus non-tg (Dunnett’s test). **E**. Representative Iba1 immunofluorescence labeling images in hippocampal areas of non-tg (2-month-old) and rTg4510 (8-month-old) mice. Scale bar = 100 µm. **F**. Semi-quantification of Iba1 signals in hippocampus from non-tg and rTg4510 mice. Positive signals of cells and processes are presented as % areas. Values are mean ± SEM. **P < 0.01, versus non-tg (Dunnett’s test). **G**. Representative Iba1 immunofluorescence labeling images in motor cortices of non-tg (2-month-old) and rTg4510 (8-month-old) mice. Scale bar = 100 µm. **H**. Semi-quantification of Iba1 signals in motor cortices from non-tg and rTg4510 mice. Positive signals of cells and processes are presented as % areas. Values are mean ± SEM. **P < 0.01, versus non-tg (Dunnett’s test). **I**. Representative AT8 immunofluorescence labeling images in hippocampal areas of rTg4510 (2- and 8-month-old) mice. Scale bar = 100 µm. **J**. Semi-quantification of AT8-positive cell numbers in hippocampus areas from rTg4510 mice. Values are mean ± SEM. **P < 0.01 and ***P < 0.001, versus non-tg (Dunnett’s test). **K**. Representative AT8 immunofluorescence labeling images in motor cortices of rTg4510 (2- and 8-month-old) mice. Scale bar = 100 µm. L. Semi-quantification of AT8-positive cell numbers in motor cortices from rTg4510 mice. Values are mean ± SEM. *P < 0.05 and ***P < 0.001, versus non-tg (Dunnett’s test).

The rTg4510 mouse line is a bigenic mouse model that permits the tetracycline-repressible over-expression of P301L mutant tau protein (Santacruz *et al.*, 2005). The expression of tau protein is controlled by the tetracycline transactivator transgene under the CaMKIIα promoter, which leads to tau accumulations in the forebrain of rTg4510 mice (Santacruz *et al.*, 2005). Intracellular deposition of tau protein was pathologically detected in the cortico-limbic area of rTg4510 mice (Ramsden *et al.*, 2005). As previously demonstrated (Ramsden *et al.*, 2005; Sahara *et al.*, 2012, 2014; Ward *et al.*, 2014), the progressions of hyperphosphorylated (AT8 immunofluorescence signals in Figs. 3 and 4I–L) and oligomeric tau accumulations increased between 4 and 8 months of age. Immunofluorescence labeling of rTg4510 mouse brains with P2Y12R, Iba1 and TSPO antibodies was conducted to characterize the microglial properties. In rTg4510 brains, immunoreactivity of P2Y12R antibody was reduced in the hippocampal CA1 and cerebral cortex as early as at 2 months of age (Figs. 3A and 4A–D, Supplemental Fig. 2). There was no obvious difference in P2Y12R signals in the midbrain (Fig. 3A) and cerebellum (data not shown) between non-tg and rTg4510 mice. Notably, P2Y12R signals were decreased by 65% at 2 months of age and by more than 90% at 8 months of age in the hippocampal CA1 and cerebral cortex of rTg4510 mice compared with non-tg mice (Fig. 4B and D). P2Y12R-positive ramified-shape microglia appeared in the whole brain areas of 8-month-old non-tg mice but not in the cortex or hippocampus of 8-month-old rTg4510 mice (Figs. 3A and 4A,C, Supplemental Fig. 2). Iba1 is thought to be one of the most reliable markers for detecting various morphological states of microglia (Imai *et al.*, 1996), and previous studies documented tau-induced neuroinflammation involving microgliosis and astrogliosis in rTg4510 mice (Yang *et al.*, 2011; Wes *et al.*, 2014; Blair *et al.*, 2015). In our study, an age-dependent increase of Iba1 immunoreactivity was observed in the cortex and hippocampus of rTg4510 mice (Figs. 3B and 4E–H). This increase was associated with hyperphosphorylated tau accumulation in rTg4510 mice but was less profound than the change in P2Y12R levels at 2–4 months of age (Figs. 3C and 4I–L). Since our previous study confirmed that the increased TSPO immunoreactivity in Iba1-positive microglia was associated with hyperphosphorylated tau accumulation in rTg4510 mice (Ishikawa *et al.*, 2018), serial sections from examined mice were labeled by TSPO antibody. The results showed that TSPO immunoreactivity was increased in the cortex and hippocampus of rTg4510 mice at 6–8 months of age (Fig. 3C), which was preceded by the diminution of P2Y12R in microglia.

**Figure 5:**
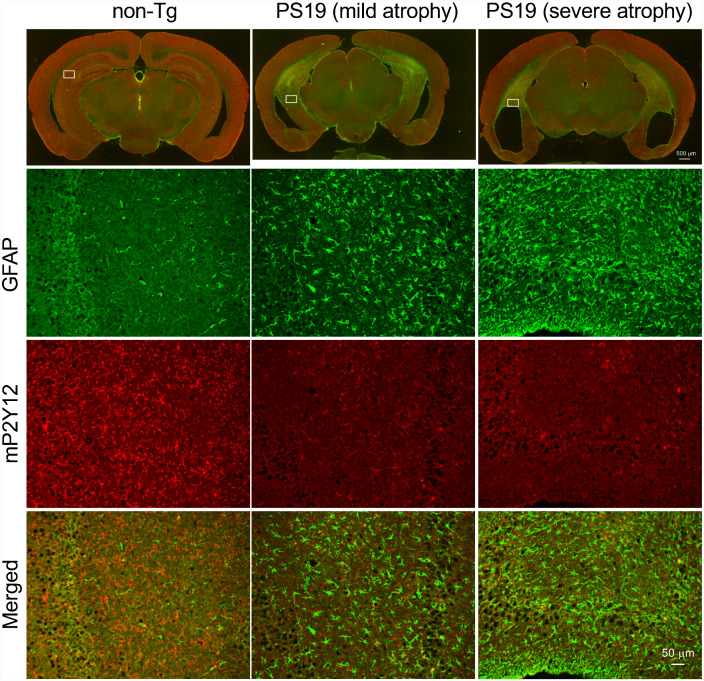
**P2Y12R immunoreactivity in the tauopathy mouse model (PS19 mice).** Upper panels: low magnification images of GFAP/P2Y12R-immunofluorescence-stained coronal brain sections (left, 21-month-old male non-tg mouse, bregma -2.3; middle, 14-month-old male PS19 mouse with mild brain atrophy; right, 11-month-old male PS19 mouse with severe brain atrophy). Due to cerebral atrophy, sections of PS19 mice were from relatively posterior levels. Scale bar = 500 µm. Second upper panels: higher magnification images of GFAP immunofluorescence labeling in hippocampal areas (boxes were indicated in low magnification images). Second lower panels: higher magnification images of P2Y12R immunofluorescence labeling in hippocampal areas. Lower panels: higher magnification images of double-immunofluorescence labeling with GFAP and P2Y12R in hippocampal areas. Scale bar = 50 µm.

To further confirm the tau pathology-dependent P2Y12R reduction, another tauopathy mouse model named PS19 was examined. PS19 mice express P301S mutant human tau under control of the mouse prion promoter (Yoshiyama *et al.*, 2007). This mouse line developed tau pathology in the hippocampus, entorhinal cortex and brain stem from 6 months of age (Maruyama *et al.*, 2013). Marked brain atrophy and ventricular enlargement were concurrent with those tau pathologies (Fig. 5). Along with the advancement of brain atrophy, P2Y12R immunoreactivity was visually decreased in the hippocampus of 11- and 14-month-old male PS19 mice (Fig. 5). In contrast, GFAP immunoreactivity was increased in parallel with the brain atrophy (Fig. 5).

### Microglia surrounding amyloid plaques of APP-based mouse models express P2Y12R at varying levels

**Figure 6:**
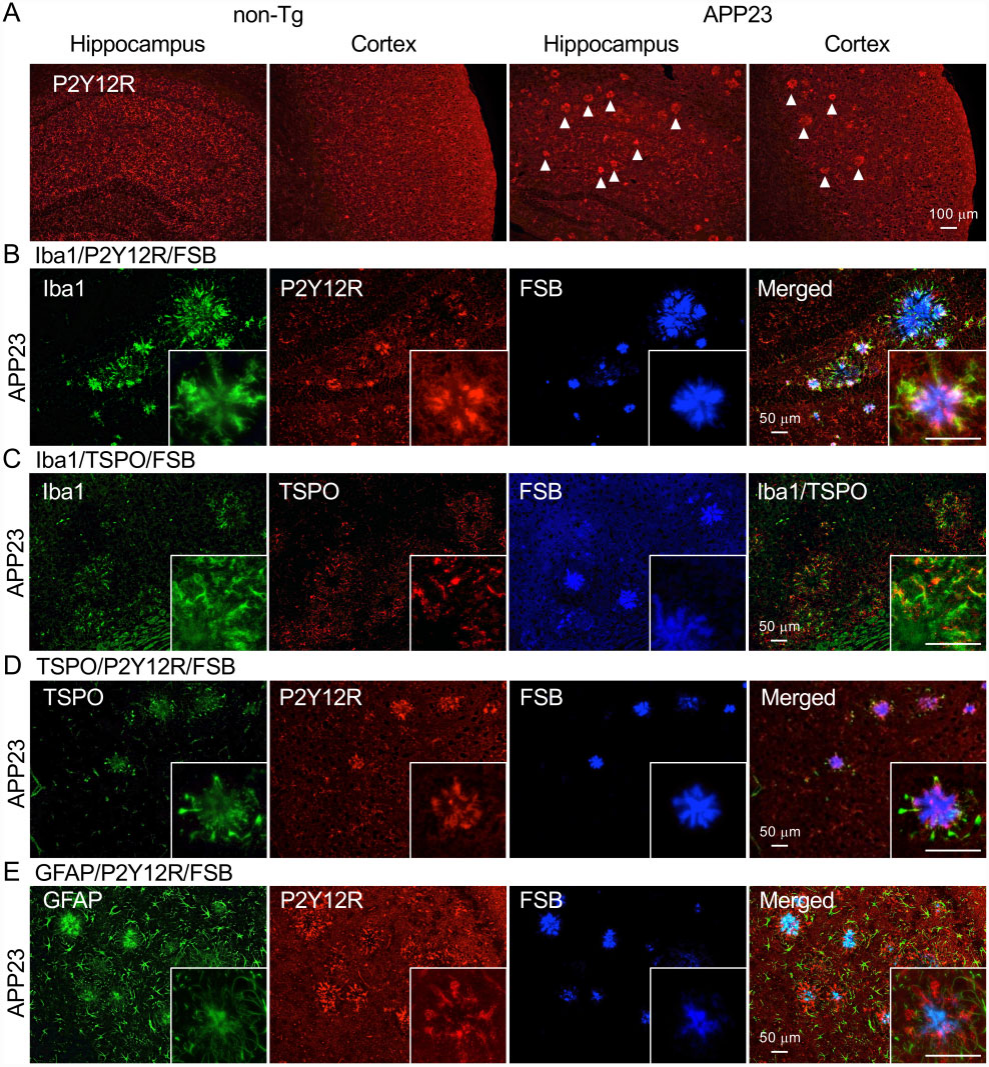
**Fluorescence labeling of anti-glial protein antibodies and FSB in the APP mouse model (APP23 mice).** **A**. Immunofluorescence labeling of anti-P2Y12R antibody in hippocampus and cortex of 22-month-old female non-tg and 28-month-old female APP23 mice. Plaque-like structures were observed in APP23 mouse by the anti-P2Y12R antibody (arrowheads). Scale bar = 100 µm. **B**. Co-labeling of Iba1 (rabbit polyclonal antibody), P2Y12R, and FSB in 28-month-old APP23 cortex. High-magnification image showed co-labeling between Iba1 and P2Y12R antibodies. **C**. Labeling of Iba1, TSPO, and FSB and merged image of Iba1/TSPO in APP23 cortex. High-magnification image showed co-labeling between Iba1and TSPO. **D**. Co-labeling of TSPO, P2Y12R, and FSB in APP23 cortex. **E**. Co-labeling of GFAP, P2Y12R and FSB in APP23 cortex. Scale bars in B–E = 50 µm.

**Figure 7:**
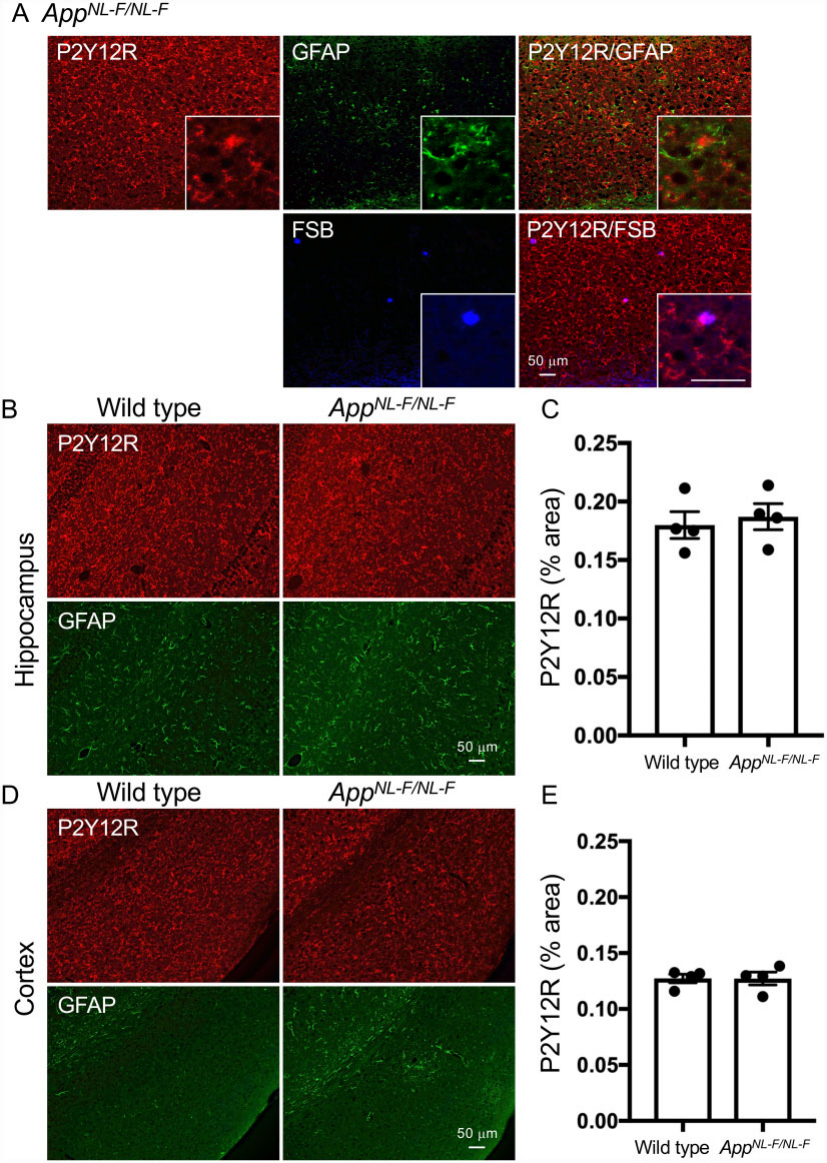
**Fluorescence labeling of P2Y12R, GFAP and FSB and quantitative analysis of P2Y12R immunoreactivity in *App^NL-F/NL-F^* and wild-type mice.** **A**. Upper panels: co-labeling of P2Y12R (red) and GFAP (green), and merged image of P2Y12R/GFAP in 18-month-old male *App^NL-F/NL-F^* cortex. Lower panels: co-labeling of P2Y12R (red) and FSB (blue). High-magnification image showed partial colocalization between P2Y12R and FSB. Scale bars = 50 µm. **B**. Representative P2Y12R (red) and GFAP (green) immunofluorescence labeling images in hippocampal areas of wild type (15-month-old) and *App^NL-F/NL-F^* (15-month-old) mice. Scale bar = 50 µm. **C**. Semi-quantification of P2Y12R signals in hippocampus from wild-type (male, *n* = 4) and *App^NL-F/NL-F^* (male, *n* = 4) mice. Positive signals of cells and processes are presented as % areas. Values are mean ± SEM. There is no significant difference between wild-type and *App^NL-F/NL-F^* mice (*P* = 0.6704, student *t*-test). **D**. Representative P2Y12R (red) and GFAP (green) immunofluorescence labeling images in cerebral cortices of wild-type (15-month-old) and *App^NL-F/NL-F^* (15-month-old) mice. Scale bar = 50 µm. **E**. Semi-quantification of P2Y12R signals in cerebral cortex from wild-type (male, *n* = 4) and *App^NL-F/NL-F^* (male, *n* = 4) mice. Positive signals of cells and processes are presented as % areas. Values are mean ± SEM. There is no significant difference between wild type and *App^NL-F/NL-F^* mice (*P* = 0.9934, student *t*-test).

It is well known that APP-based transgenic mice recapitulate the amyloid pathology without NFT formation. The transgenic mouse model APP23 expresses human APP with the Swedish mutation (Sturchler-Pierrat *et al.*, 1997). These mice develop amyloid plaques in the cerebral cortex and hippocampus with aging. Moreover, there is a tight association of activated microglia detected by an anti-Mac-1 antibody with congophilic dense-core amyloid plaques in this model (Stalder *et al.*, 1999; Bornemann *et al.*, 2001). In the present study, the periphery of plaque-like structures was positively labeled by the anti-P2Y12R antibody in a 28-month-old female APP23 mouse hippocampus and cerebral cortex (Fig. 6A). We also observed that Iba1, TSPO and GFAP signals encompassed dense-core plaques (Fig. 6B–E). TSPO immunoreactivities were localized in Iba1-positive microglia (Fig. 6C). However, most TSPO and GFAP signals did not colocalize with the P2Y12R signal by visual observation (Fig. 6D and E). To further assess the presence of P2Y12R around amyloid plaques, we examined *App^NL-F/NL-F^* mice that produce a humanized Aβ peptide with an enhanced yield of a more amyloidogenic subspecies, Aβ_42_ (Saito *et al.*, 2014). At 18 months of age, *App^NL-F/NL-F^* mice developed compact plaques in the cerebral cortex and hippocampus (Fig. 7A). P2Y12R-positive microglia and GFAP-positive astrocytes constantly existed in *App^NL-F/NL-F^* mouse brain regions with amyloid pathology but were not overtly associated with these plaques, unlike dense-core plaques in APP23 mice (Fig. 7). Importantly, P2Y12R immunoreactivities in the cerebral cortex and hippocampus of 15-month-old male *App^NL-F/NL-F^* mice had similar levels to those in age-matched male wild-type mice (Fig. 7B–E). These results were in sharp contrast to the robust reduction of P2Y12R levels in tauopathy mouse models.

### In vitro [^11^C]AZD1283 autoradiography in PS19 and APP-KI mouse brains

**Figure 8:**
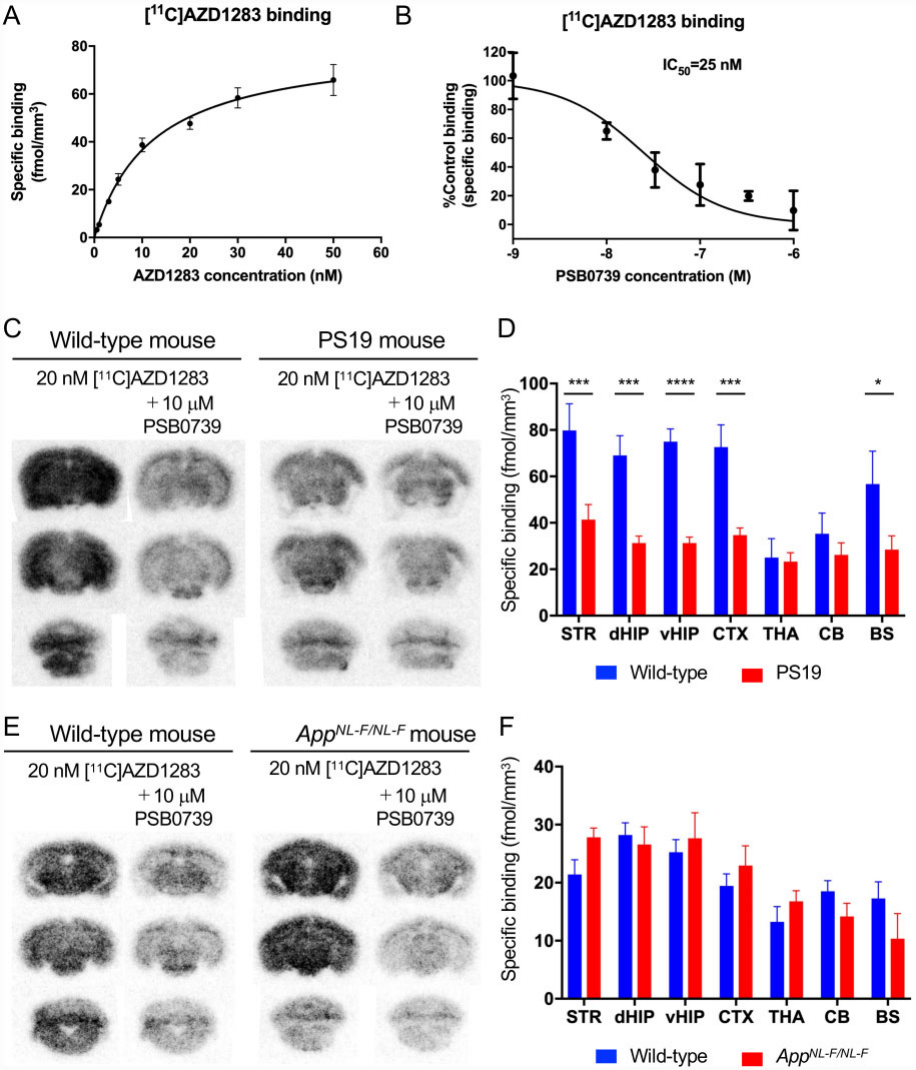
**In vitro autoradiography of radio-labeled P2Y12R antagonist.** **A**. Signal intensities in C57BL/6J mouse cerebral cortex were plotted to the concentrations of [^11^C]AZD1283 (0.5, 1, 3, 5, 10, 20, 30, 50 nM). Saturation curve was fitted by one site-specific binding. Error bars: SEM. **B**. Competition binding assay of [^11^C]AZD1283 autoradiography was performed with non-radioactive PSB0739. Radioisotope signals were obtained from incubation with 20 nM [^11^C]AZD1283 and PSB0739 (1,10, 33, 100, 330, 1000 nM). Error bars: SEM. **C**. Representative [^11^C]AZD1283 autoradiograms of coronal sections from non-tg (wild-type) and PS19 mice. Brain sections were incubated with 20 nM [^11^C]AZD1283 in the absence or presence of 10 µM PSB0739. **D**. Specific binding (fmol/mm^3^) of [^11^C]AZD1283 in brain regions (striatum, STR; dorsal hippocampus, dHIP; ventral hippocampus, vHIP; cerebral cortex, CTX; thalamus, THA; cerebellum, CB; brain stem, BS) of wild-type (6- to 12-month-old, 2 males and 3 females) and PS19 (6- to 12-month-old, 10 males and 5 females) mice. Values are mean ± SEM. *P < 0.05 in BS, ***P < 0.001 in STR, dHIP and CTX, ****P < 0.0001 in vHIP, and *P* > 0.9999 in THA and CB (Bonferroni’s comparisons test). **E**. Representative [^11^C]AZD1283 autoradiograms of coronal sections from wild-type and *App^NL-F/NL-F^* mice. Brain sections were incubated with 20 nM [^11^C]AZD1283 in the absence or presence of 10 µM PSB0739. F. Specific binding (fmol/mm^3^) of [^11^C]AZD1283 in brain regions (STR, dHIP, vHIP, CTX, THA, CB, BS) of wild-type (12-month-old, female, *n* = 8) and *App^NL-F/NL-F^* (13-month-old, female, *n* = 8) mice. Values are mean ± SEM. *P* = 0.5646 in BS, *P* = 0.7686 in STR, *P* > 0.9999 in dHIP, vHIP, CTX, THA and CB (Bonferroni’s comparisons test).

A candidate drug for thrombosis, AZD1283 (Supplemental Fig. 3), is one of the ethyl 6-aminonicotinate acyl sulfonamides that are potent antagonists of P2Y12R (Bach *et al.*, 2013). To develop a radiotracer for P2Y12R, [^11^C]AZD1283 was synthesized, and its specific binding to P2Y12R in brain tissues was quantified. *K*_d_ (ligand concentration that binds to half the receptor sites at equilibrium) and *B*_max_ (maximum number of binding sites) on the C57BL/6J mouse cerebral cortex were examined by [^11^C]AZD1283 autoradiography (Fig. 8A) and were determined as 12.2 ± 2.04 nM and 81.25 ± 5.04 fmol/mm^3^, respectively. To examine the specificity of the radioligand binding for P2Y12R, the level of specific binding of [^11^C]AZD1283 was determined in the presence of a range of concentrations of non-radioactive PSB0739, a potent P2Y12R antagonist (Fig. 8B), and the concentration of PSB0739 inducing 50% inhibition of the radioligand binding (IC_50_) was quantified as 25 nM. The specific binding of [^11^C]AZD1283 was then examined in the brains of the model mice. In comparison with wild-type C57BL/6J mice, PS19 mice at 6–12 months of age have fewer binding sites in the striatum, dorsal hippocampus, ventral hippocampus, cerebral cortex, and brain stem (Fig. 8C and D). Two-way ANOVA analysis (Fig. 8D) showed that tracer binding was influenced by both genotype (*F*(1,124) = 61.32, *P* < 0.0001) and brain region (*F*(6,124) = 7.785, *P* < 0.0001), and that genotype differentially affected the tracer binding in brain areas (*F*(6,124) = 2.943, *P* < 0.05). On the other hand, [^11^C]AZD1283 binding on *App^NL-F/NL-F^* and APP23 mice was similar to wild-type C57BL/6J mice (Fig. 8E and F, Supplemental Fig. 4). As shown in Fig. 8F, tracer bindings were not different between wild-type and *App^NF-L/NF-L^* mice (*F*(1,98) = 0.06775, *P* = 0.7952), but were significantly different between brain regions (*F*(6,98) = 8.259, *P* < 0.0001). Genotype did not affect the tracer binding in brain areas (*F*(6,98) = 1.498, *P* = 0.1869). These data support the immunohistochemical observations on the P2Y12R levels in PS19, *App^NL-F/NL-F^*, and APP23 mouse brains.

## Discussion

The implication of microglia in AD and related disorders has recently attracted attention in terms of achieving effective therapies by the use of neuroinflammatory targets. In this study, we investigated the microglial response in AD mouse models. Our previous studies demonstrated in vivo TSPO-PET imaging to verify TSPO-positive microglial activation in mouse models with Aβ or tau pathology (Maeda *et al.*, 2011; Ishikawa *et al.*, 2018). The present study revealed that immunoreactivity of P2Y12R was regressed in tauopathy mouse models before massive accumulations of intraneuronal tau deposits and an elevation of TSPO immunoreactivity (Figs. 3 and 4). The reduction of P2Y12R in association with tau pathologies was also observed in both human AD and SD-NFT entorhinal cortices (Fig. 1). These data suggest that the progression of tau pathology strongly reflects the microglial transition from homeostatic phenotype to DAM phenotype. On the other hand, immunoreactivities of Iba1, TSPO, P2Y12R and GFAP were accumulated around dense-core plaques in APP23 mice (Fig. 6). Since prominent gliosis was involved in AD pathogenesis as a result of neuroinflammatory response (Minter *et al.*, 2016; Ransohoff, 2016), increases of TSPO and GFAP levels apparently exhibit reactive gliosis for a plaque formation. However, our study indicated that P2Y12R also existed in surrounding plaques in APP mice (Figs. 6 and 7).

Most APP mouse models recapitulate the amyloid pathology in AD patients, but fail to develop tau pathologies (Sasaguri *et al.*, 2017). Although extracellular Aβ deposits are common features of both APP mouse models and AD patients, the composition of Aβ plaques in APP-overexpressing mouse models (e.g. Tg2576, APP23) was different from amyloid plaques in AD patients (Saito *et al.*, 2011). When microglial gene expression was examined by qPCR analysis, the expression pattern in the APP/PS1 model differed from that observed in AD patients (Navarro *et al.*, 2018). These differences can be explained by a different kinetics of the Aβ accumulation. The microglial response to the fast, extensive Aβ accumulation in APP mouse models differs from the chronic Aβ accumulation with longer life span in human brains. In our study, the difference in microglial status between APP mouse models and AD patients was also observed. The P2Y12R levels in APP mouse models were almost equivalent to those in wild-type mice (Figs. 6–8 and Supplemental Fig. 4), although different P2Y12R immunoreactivity around amyloid plaques in the two APP mouse models was observed (Figs. 6 and 7). In AD patients, P2Y12R immunoreactivity was decreased in comparison to healthy controls (Fig. 1A). Unchanged P2Y12R levels may be due to less microglial activation in APP mouse models than that in AD patients, as mouse models do not recapitulate the extensive neuronal loss observed in AD patients. Nevertheless, further study will be needed to define the primary event causing microglial status change.

Several transgenic mouse lines expressing human mutant tau have demonstrated age-dependent pathological tau accumulation and associated neuronal loss (Roberson, 2012). Although it is still unresolved whether pathological tau induces aberrant neuroinflammation or microgliosis precedes NFT formation, both gliosis and neuroinflammation are prevalent in human tauopathies and mouse models (Leyns and Holtzman, 2017). Recent in vivo imaging studies with TSPO-PET demonstrated increased TSPO signals in both human tauopathies and mouse models (Miyoshi *et al.*, 2010; Maeda *et al.*, 2011; Zhang, 2015; Ishikawa *et al.*, 2018). In rTg4510 mice, studies using PET imaging and immunohistochemistry confirmed that age-dependent TSPO accumulation followed pathological tau accumulation and brain atrophy (Ishikawa *et al.*, 2018; Sahara *et al.*, 2018). To further investigate the temporal change of microglial phenotype during the development of tauopathy, we examined the morphological and molecular characteristics by using antibodies against several microglial markers (e.g. Iba1, TSPO, P2Y12R) in rTg4510 brains (Fig. 3). Iba1 staining in rTg4510 mice showed age-dependent increases of unramified microglial cells in the cerebral cortex and hippocampus. Immunostaining with TSPO antibody further confirmed that intraneuronal tau accumulations lead the TSPO expression in rTg4510 mice. In this sense, microglial activation was secondarily induced by neuronal tau depositions. However, primary microglial activation cannot be excluded because detection of TSPO is insufficient for evaluating microglial activation. To track down the initial step of microglial activation, additional benchmarks will be needed. Since P2Y12R declines preceded increases of Iba1 and TSPO signals in rTg4510 mice, P2Y12R would be a complemental marker for evaluating microglial activation.

Immunohistochemical examinations in two distinct tauopathy mouse models revealed the reduction of P2Y12R in human P301L/P301S tau-expressing brain regions long before pathological tau accumulation (Figs. 2–5). These data suggest that most homeostatic microglia were probably transformed into non-homeostatic microglia in tauopathy mouse models when the P2Y12R expression declined. Our recent microglial gene expression analysis in neurodegenerative mouse models including *App^NL-G-F/NL-G-F^*, rTg4510 and SOD^G93A^ mice confirmed that DAM genes were uniformly unregulated in all three mouse models (Sobue *et al*., 2021). Therefore, a neurodegenerative phenotype of microglia in rTg4510 mice was similar to the original DAM signature defined by RNA-seq analysis in 5XFAD mice (Keren-Shaul *et al.*, 2017). Since the microglial transition in tauopathy mouse models occurred earlier than expected, non-pathological neuronal dysfunctions instead of filamentous tau aggregations may have triggered the microglial activation. Meanwhile, neuroprotective functions of microglia through somatic microglia-neuron interactions were reported (Cserep *et al.*, 2020). In that study, microglial processes contacted directly with neuronal cell bodies, an interaction that is highly dependent on the existence of P2Y12R. Purinergic signaling from neuronal mitochondria was involved in the somatic microglia-neuron interactions. Thus, P2Y12R clustering in the microglial process is likely dependent on neuronal mitochondrial activity. Assumingly, excessive tau proteins may affect purinergic signaling via the mitochondrial impairment leading to the reduction of P2Y12R clustering. Mitochondrial distribution deficits in rTg4510 neuronal cell somas observed by array tomography (Kopeikina *et al.*, 2011) strongly support the excessive tau-induced mitochondrial abnormalities. Since human P301L tau in 2-month-old rTg4510 mice was less phosphorylated (Sahara *et al.*, 2012), mitochondrial activities (e.g. ATP generation, Ca^2+^ signaling) might be disrupted by non-aggregated tau species. Subsequently, neuronal protection by P2Y12R clustering of microglial processes was likely extinguished in the early stage of tauopathy.

Neuroinflammation, an inflammatory response in CNS, is mediated by the production of cytokines, chemokines, reactive oxygen species, and secondary messengers (reviewed in DiSabato *et al.*, 2016). Aspects of neuroinflammation vary within the context of disease, injury, infection or stress. Inflammation usually resolves itself, but it can be prolonged, and pathologic inflammation can occur, resulting in detrimental effects on brain function due to excessive or persistent release of cytotoxic factors. The chronic over-activation of pro-inflammatory response has been implicated in many neurodegenerative disorders including AD (reviewed in Chaney *et al.*, 2019). In vivo visualization of neuroinflammation is necessary to understand its contribution to the initiation and progression of AD. Because the early phase of microglial activation during the process of AD pathogenesis is hardly detectable by current in vivo imaging techniques (Narayanaswami *et al.*, 2018; Chaney *et al.*, 2019), novel PET radiotracers for imaging neuroinflammation are urgently needed. Among neuroinflammation targets (e.g. TSPO, glycogen synthase kinase 3, monoamine oxidase-B, chemokine receptor CX3CR1, purinergic receptors P2X7R and P2Y12R), P2Y12R is a potent marker for visualizing homeostatic microglial functions by PET imaging, although positron-labeled P2Y12R antagonists have not yet been reported. As potential candidates, reversible competitive antagonists have been synthesized (Abbracchio *et al.*, 2006; Chaney *et al.*, 2019). However, due to their lower lipophilicities and higher molecular weights, these molecules seem to have difficulty crossing the blood-brain barrier. In fact, due to the lower lipophilicity of AZD1283 (LogD_pH7.4_=1.4), PET imaging showed no uptake signal of [^11^C] AZD1283 in the wild-type mouse brain (Supplemental Fig. 3). Despite being useless for PET imaging, the binding specificity of [^11^C]AZD1283 to P2Y12R was confirmed by ARG. Thus, development of desirable radioligands derived from this ligand with higher lipophilicity could be possible in the future.

In this study, microglial phenotypic changes during the process of neurodegeneration associated with tau and Aβ pathologies were demonstrated by tracking microglial markers. Most importantly, mouse model studies revealed that reduction of P2Y12R immunoreactivity was strongly associated with tau pathology while amyloid plaques do not decrease P2Y12R signals, although biological variables from sex differences still need to be investigated. A decline of P2Y12R-positive microglia in tauopathy mouse models was found much earlier than intracellular filamentous tau accumulation. Human AD brains, different from APP mice, show less P2Y12R immunoreactivity in the regions of both tau and amyloid pathologies. In agreement with this observation, Walker et al. confirmed that P2Y12R immunoreactivity in human AD cortical layers was significantly lower than those in a low plaque non-demented case and a high plaque non-demented case (Walker *et al.*, 2020). Interestingly, in that study, many diffuse plaques showed colocalization with P2Y12R-positive microglia, but not with mature-type cored plaques (Walker *et al.*, 2020). If these diffuse plaques in human brains are similar to plaques in APP mice, common signals in both humans and mice must exist to sequester P2Y12R-positive microglia. Since P2Y12R-positive microglia surrounding plaques are morphologically different from ramified microglia, plaque-associated P2Y12R-positive microglia may be distinct from homeostatic microglia. Future studies will be required to characterize subtypes of P2Y12R-positive microglia. Nevertheless, P2Y12R would be a marker applied enthusiastically for the detection of neuroinflammatory responses to neurodegenerative processes associated with either tau or Aβ pathology.

## Supplementary Material

fcab011_Supplementary_Data

## Abbreviations

A =: amyloid-β
AD =: Alzheimer’s disease
ADP =: adenosine diphosphate
APP =: amyloid precursor protein
CNS =: central nervous system
DAM =: disease-associated microglia
FSB =: 1-fluoro-2,5-bis(3-hydroxycarbonyl-4-hydroxystyryl)benzene
IL-1: β =interleukin-1β
IRB =: Institutional Review Board
NTFs =: neurofibrillary tangles
P2Y12R =: P2Y12 receptor
SD-NFT =: senile dementia of NFT type
SDS-PAGE =: sodium dodecyl sulfate-polyacrylamide gel electrophoresis
TSPO =: mitochondrial 18-kDa translocator protein
