# Supplementary material for: Distinct microglial response against Alzheimer's amyloid and tau pathologies characterized by P2Y12 receptor
Source: Brain Commun. 2021 Jan 29;3(1):fcab011. doi: 10.1093/braincomms/fcab011 (PMC7901060; doi:10.1093/braincomms/fcab011)
Supplement: fcab011_Supplementary_Data [file fcab011_supplementary_data.pdf]

Supplemental Figure 1

A normal control SD-NFT

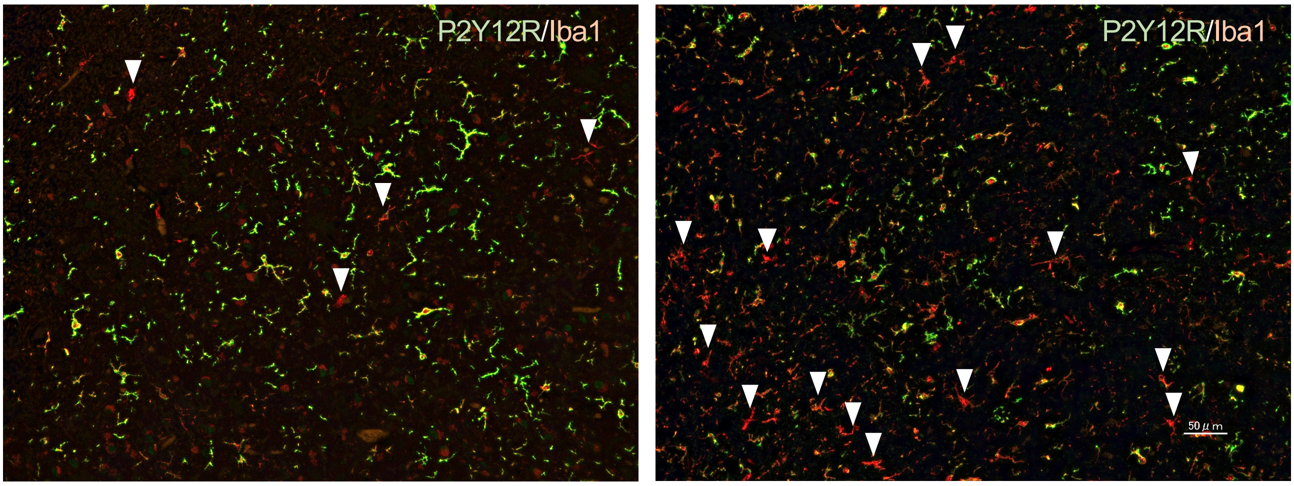

B Iba1-positive P2Y12R-negative cells

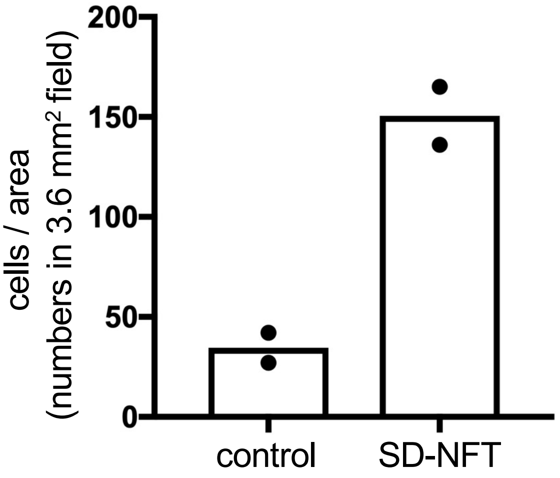

Supplemental Figure 1. Semi-quantitative analysis of Iba1-positive and P2Y12R-negative microglia in the hippocampal formation of human subjects. A. Representative images of P2Y12R (green) / Iba1 (red) double-staining in the hippocampal region from normal control (92-year-old, female, non-demented subject, Braak NFT: II, CERAD plaque: 1) and SD-NFT (96-year-old, female, Braak NFT: II~III, CERAD plaque: 0) subjects. Inverted triangles show Iba1-positive and P2Y12R-negative microglia. Scale bar = 50 μm. B. Averaged cell numbers in the area of hippocampal formation from control and SD-NFT subjects. For counting Iba1-positive and P2Y12R-negative microglia, 10 rectangular fields (0.36 mm^2) from each subject were randomly examined. The numbers of Iba1-positive and P2Y12R-negative microglia in two controls and two SD-NFT subjects were 27, 42, 136 and 165, respectively.

Supplemental Figure 2

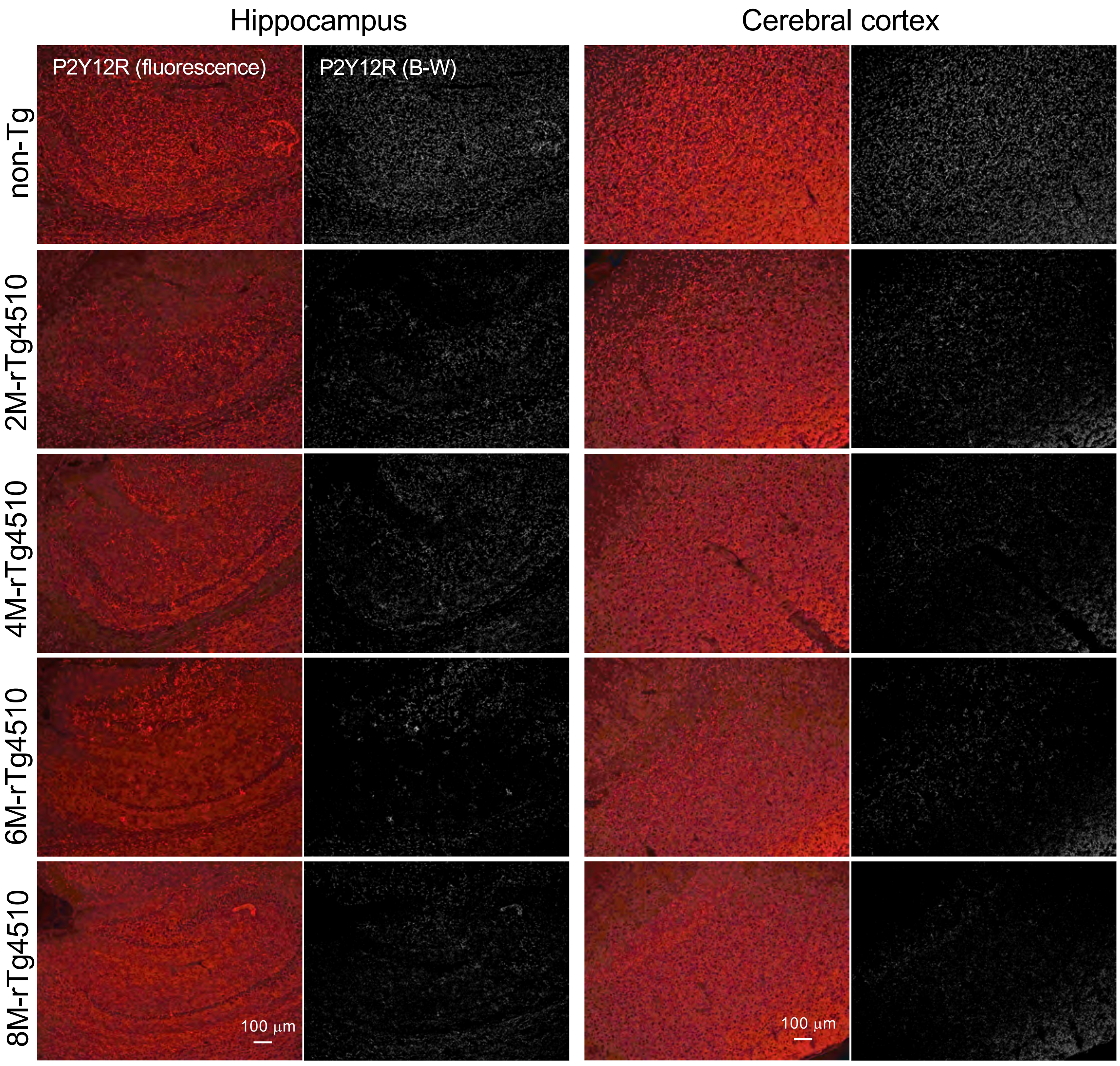

Supplemental Figure 2. P2Y12R immunofluorescence signals in tauopathy mouse brains. Representative images of hippocampi and cerebral cortices from 2-month-old non-tg, 2-month-old rTg4510, 4.1-month-old rTg4510, 6.7-month-old rTg4510, and 8.1-month-old rTg4510 mice. The next images to the right were changed to black and white. Intense signals (white color) in rTg4510 mice were significantly decreased with age. Scale bars = 100 μm.

Supplemental Figure 3

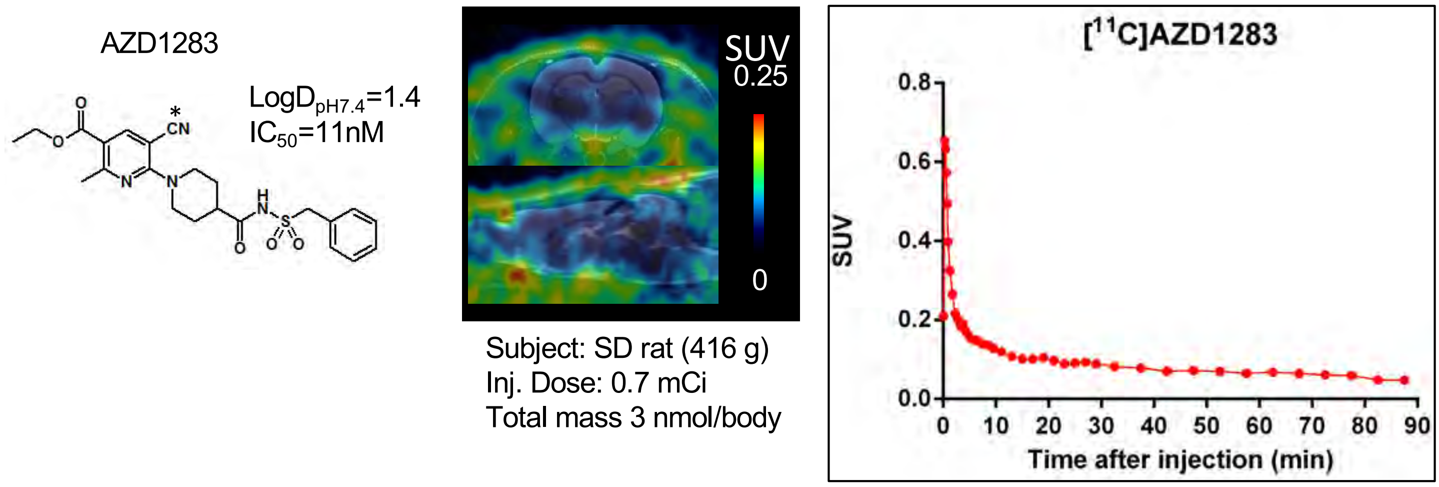

Supplemental Figure 3. [^{11}C]AZD1283 signal uptake in SD rat by in vivo PET imaging. Left: chemical structure of [^{11}C]AZD1283. Middle: [^{11}C]AZD1283 PET image of SD rat generated by averaged dynamic scan data at 0-90 min. MRI image was overlaid for spatial alignment. Right: [^{11}C]AZD1283 signal kinetics of SD rat brain.

Supplemental Figure 4

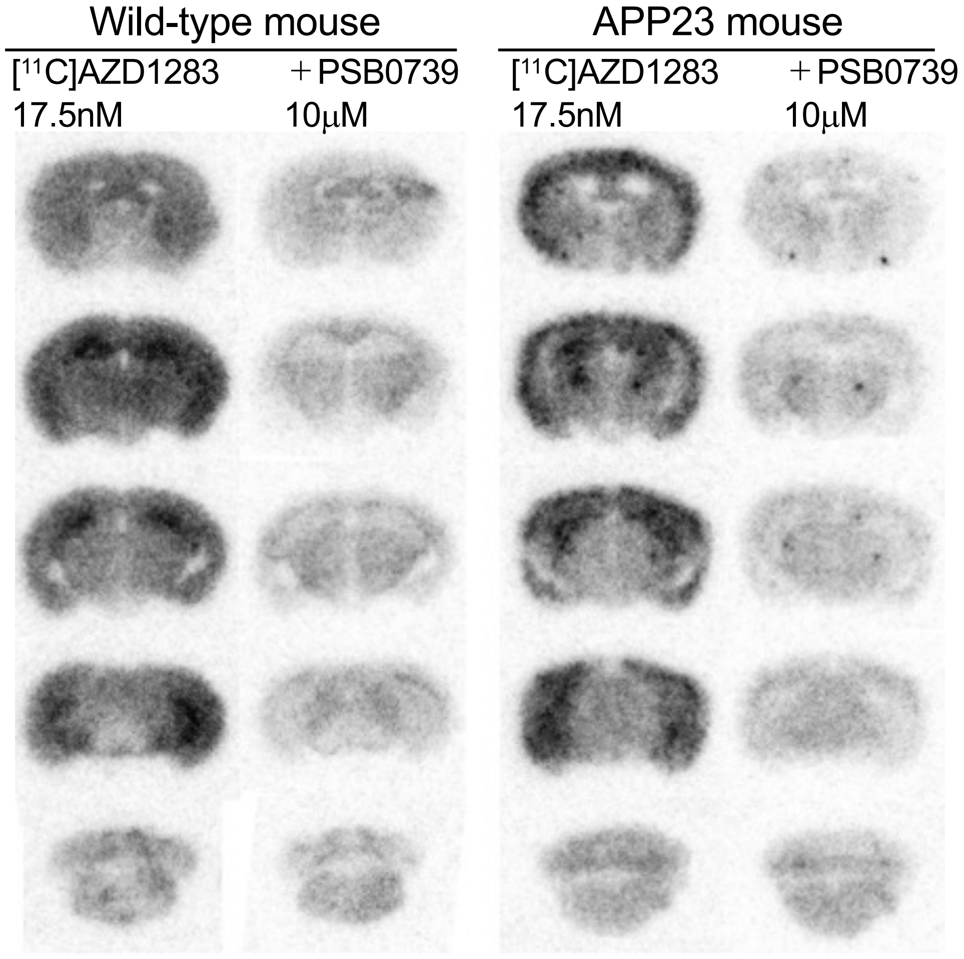

Supplemental Figure 4. [^{11}C]AZD1283 autoradiograms of coronal sections from non-tg (wild-type) and APP23 mice. Brain sections were incubated with 17.5 nM [^{11}C]AZD1283 in the absence or presence of 10 μM PSB0739.
